# Urinary c‐peptide creatinine ratio (UCPCR) as a predictor of coronary artery disease in type 1 diabetes mellitus

**DOI:** 10.1002/edm2.413

**Published:** 2023-02-20

**Authors:** Rana Irilouzadian, Siamak Afaghi, Farzad Esmaeili Tarki, Fatemehsadat Rahimi, Nasser Malekpour Alamadari

**Affiliations:** ^1^ Burn Research Center Iran university of medical sciences Tehran Iran; ^2^ Prevention of Metabolic Disorders Research Center, Research Institute for Endocrine Sciences Shahid Beheshti University of Medical Sciences Tehran Iran; ^3^ Research institute of internal medicine Shahid Modarres hospital, Shahid Beheshti university of medical sciences Tehran Iran; ^4^ Chronic Respiratory Diseases Research Center National Research Institute of Tuberculosis and Lung Diseases, Masih Daneshvari Hospital, Shahid Beheshti University of Medical Sciences Tehran Iran; ^5^ Department of Surgery, Clinical Research and Development Center, Shahid Modarres Hospital Shahid Beheshti University of Medical Sciences Tehran Iran

**Keywords:** coronary artery disease, C‐peptide, risk factor, type 1 diabetes mellitus, urinary C‐peptide creatinine ratio

## Abstract

**Background:**

Elevated C‐peptide has been suggested as a risk factor for coronary artery disease (CAD). Elevated urinary C‐peptide to creatinine ratio (UCPCR) as an alternative measurement is shown to be related to insulin secretion dysfunction; however, data regarding UCPCR predictive value for CAD in diabetes mellitus (DM) are scarce. Therefore, we aimed to assess the UCPCR association with CAD in type 1 DM (T1DM) patients.

**Methods:**

279 patients previously diagnosed with T1DM included and categorized into two groups of CAD (*n* = 84) and without‐CAD (*n* = 195). Furthermore, each group was divided into obese (body mass index (BMI) ≥ 30) and non‐obese (BMI < 30) groups. Four models utilizing the binary logistic regression were designed to evaluate the role of UCPCR in CAD adjusted for well‐known risk factors and mediators.

**Results:**

Median level of UCPCR was higher in CAD group compared to non‐CAD group (0.07 vs. 0.04, respectively). Also, the well‐acknowledged risk factors including being active smoker, hypertension, duration of diabetes, and body mass index (BMI) as well as higher levels of haemoglobin A1C (HbA1C), total cholesterol (TC), low‐density lipoprotein (LDL) and estimated glomeruli filtration rate (e‐GFR) had more significant pervasiveness in CAD patients. Based on multiple adjustments by logistic regression, UCPCR was a strong risk factor of CAD among T1DM patients independent of hypertension, demographic variables (gender, age, smoking, alcohol consumption), diabetes‐related factors (diabetes duration, FBS, HbA1C), lipid profile (TC, LDL, HDL, TG) and renal‐related indicators (creatinine, e‐GFR, albuminuria, uric acid) in both patients with BMI≥30 and BMI < 30.

**Conclusion:**

UCPCR is associated with clinical CAD, independent of CAD classic risk factors, glycaemic control, insulin resistance and BMI in type 1 DM patients.

## Graphical abstract

Urinary C‐peptide to creatinine ratio (UCPCR) is associated with both insulin secretion disturbance and insulin resistance. Among type 1 diabetes mellitus (T1DM) patients, the mean UCPCR is significantly higher in CAD group compared to non‐CAD group. UCPCR is independently associated with CAD. UCPCR leads to a similar risk of CAD in both obese (>30 kg/m2) and non‐obese (<30 kg/m2) patients with T1DM.

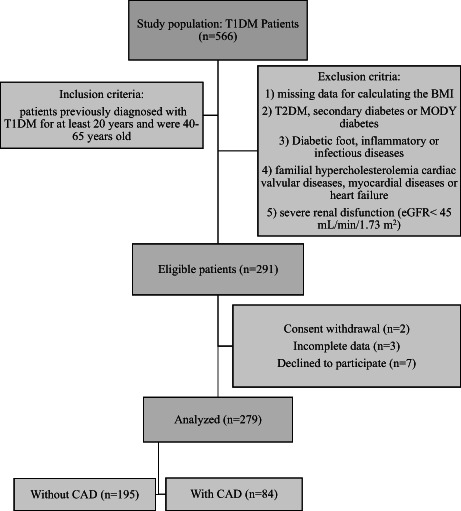

## 1 INTRODUCTION

Type 1 diabetes mellitus (T1DM), which is a deficient production or dysfunction of insulin, has become a worldwide and provincial health concern with a significantly increasing incidence.^1^ Cardiovascular diseases (CVD) are recognized as the main etiologic factor for mortality in T1DM patients even in those with appropriate metabolic control.^2^ Insulin is an endogenous, anabolic peptide hormone secreted from pancreatic beta cells and regulates blood glucose homeostasis. Pre‐proinsulin is synthesized by pancreatic beta cells and rapidly converts to proinsulin, an 86 amino acid polypeptide which is then cleaved to generate C‐peptide and insulin equimolarly, containing 31 and 51 amino ac ids, respectively.^3^, ^4^, ^5^ Insulin with a half‐life of 3–5 min is metabolized in liver by approximately 50% and has a variable clearance in peripheral circulation. Therefore, its direct measurement is not an accurate method for assessing pancreatic beta cell's function. Also, blood insulin test may have cross‐reactivity with exogenous insulin in patients with diabetes who take insulin. Contrarily, C‐peptide is slightly extracted by liver but cleared mainly in kidneys and about 5% of total produced C‐peptide is excreted into the urine.^6^, ^7^ As a result, C‐peptide can be spotted in serum and urine. Serum C‐peptide test is an inconvenient method due to difficult sample collection and can be measured in a fasted or non‐fasted sample and after stimulation.^6^, ^8^, ^9^ It is also falsely elevated in patients with renal impairment.^10^, ^11^ C‐peptide is mostly used to distinguish between insulinoma and factitious hypoglycaemia in nondiabetic individuals.^7^ It has been demonstrated that C‐peptide is associated with duration of diabetes, age of diagnosis, the need for insulin therapy, predicting glycaemic control, pro‐inflammatory condition, and microvascular and macrovascular complications. Elevated C‐peptide level is related to insulin resistance and metabolic syndrome. Accordingly, higher levels of C‐peptide have been suggested as risk factor of coronary artery disease (CAD) and death in patients without type 2 diabetes mellitus (T1DM).^12^, ^13^, ^14^, ^15^

Urinary C‐peptide is a non‐invasive and practical test with shortcomings in patients with renal impairment.^6^, ^7^ Urinary C‐peptide creatinine ratio (UCPCR) is another simple and reliable test that is correlated with serum C‐peptide and 24‐h urinary C‐peptide even in moderate renal impairment.^9^, ^16^, ^17^ However, the amount of UCPCR varies in different genders and weights as a result of alteration in muscle mass and creatinine levels.^18^ The role of UCPCR in classification of different types of diabetes has been well established.^7^, ^19^ UCPCR has been shown to relate to insulin resistance in patients without diabetes and obese children^20^, ^21^ but the relation of UCPCR to CAD in individuals with type 1 diabetes mellitus (T1DM) has not been evaluated. Therefore, in the present study we have aimed to investigate roles of UCPCR in CAD in T1DM patients classified by BMI.

## 2 METHODS

### 2.1 Study design and participants

**FIGURE. 1:**
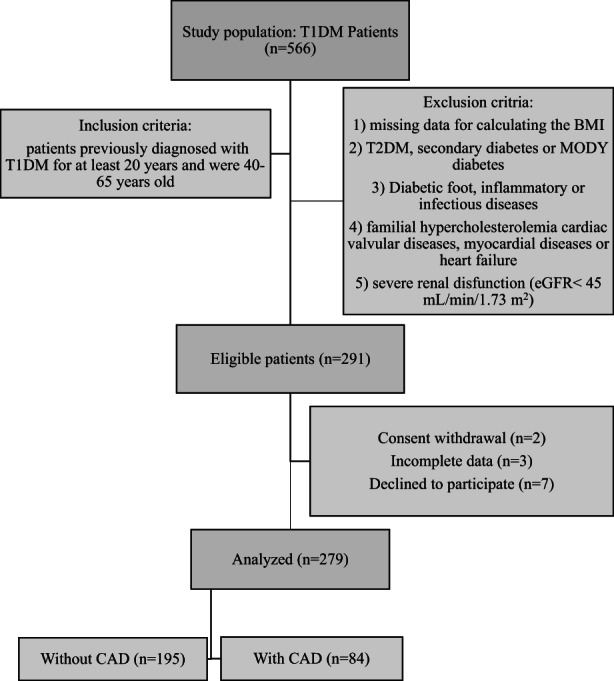
Flow chart of the studied groups. BMI, body mass index; CAD, coronary artery disease; DM, diabetes mellitus; eGFR, estimated glomerular filtration rate; MODY, maturity onset diabetes of young; T1DM, type 1 diabetes mellitus; T2DM, type 2 diabetes mellitus.

The present research has enrolled 279 patients with a T1DM diagnosis at Shahid Modarres Hospital affiliated to Shahid Beheshti University of Medical Sciences in Tehran, Iran, from November 2020 to November 2021 (Figure 1). The exclusion criteria were as follows: patients with (1) missing information needed for BMI calculation; (2) Type 2 DM, MODY diabetes or secondary diabetes, (3) Diabetic foot, inflammatory or infectious diseases; (4) heart failure, cardiomyopathy, myocarditis, cardiac valvular diseases, or familial hypercholesterolemia; and (5) renal disfunction (eGFR<45 mL/min/1.73 m^2^) due to its effect on C‐peptide urinary excretion. Finally, a total of 279 subjects (84 patients with CAD and 195 patients without CAD) have been permitted for evaluation in the present study. Written informed consent has been acquired from patients, and the study has been given approval by the ethics review board of the Shahid Beheshti University of Medical Sciences (Ethical code number: IR.SBMU.MSP.REC.1400.083).

### 2.2 Data measurements

The individuals' demographic features, habits, and past medical histories were collected using the hospital's computerized patient record system. The body mass index (BMI) was calculated by the weight divided by the square of the height (kg/m2). Blood pressure (BP) has been recorded three times consecutively, on the left arm following the seated condition of patient lasting for at least 5 min. Consequently, the average of three recorded BPs was employed for evaluations. Blood samples have been taken after 8‐hour fasting and before the breakfast. Fasting glucose, low‐density lipoprotein cholesterol (LDL‐C), high‐density lipoprotein cholesterol (HDL‐C), triglycerides (TGs), total cholesterol (TC), creatinine (Cr), and uric acid (UA) were quantified. High‐performance liquid chromatography was used to determine HbA1C levels. A chemiluminescence immunoassay analyser has been used to detect C‐peptide and blood insulin levels. An immunoturbidimetric test analyser has been employed to detect albuminuria, and the e‐GFR has been determined by the Chronic Kidney Disease Epidemiology Collaboration (CKD‐EPI) equation and creatinine levels. Patients collected urine specimen at the time of blood sampling and the urinary C‐peptide and creatinine were analysed using electrochemiluminescence immunoassay and Roche P800 platform, respectively, to calculate UCPCR (nmol/mmol).

### 2.3 Definitions

A BMI ≥30 kg/m^2^ has been described as obesity, based on the criteria of the World Health Organization (WHO) obesity classification^22^; hence, the categorization of patients based on BMI was performed into 2 groups of non‐obese patients (BMI ≤30 kg/m^2^) and obese (BMI ≥30 kg/m^2^). Diabetic cases who had already been identified were discovered by an examination of their health records using the WHO criteria from 1999: random blood glucose levels of more than 200 mg/dL (11.1 mmol/L), Fasting blood glucose levels of more than 126 mg/dL (7.0 mmol/L), with clinical manifestations (diabetic ketoacidosis, polydipsia polyuria), or an abnormal oral glucose tolerance test result. Abnormal glycaemia must be presented on two separate occasions if the patient had absence of symptoms. The current use of antihypertensive therapy or a blood pressure of 140/90 mmHg or was considered hypertension. The following criteria were used to diagnose CAD in the Department of Cardiology at Shahid Modarres Hospital in Tehran, Iran: (1) ischemia in the ECG, (2) Angina and (3) hard CAD. Angiography‐shown coronary artery blockage of 50% or more, coronary revascularization, or a history of myocardial infarction (MI) verified by Q waves evident in the ECG or medical records was considered hard CAD.

### 2.4 Statistical analysis

Categorical variables and continuous data having a normal distribution have been presented as percentages (%) and the mean ± standard deviation (SD), respectively. The chi‐squared test and Student's *t* test were utilized to evaluate differences between the two groups, while one‐way ANOVA has been utilized for three groups or more. To compare continuous data, the Mann–Whitney *U*‐test was utilized. In T1DM patients, the risk variables for CAD were determined using binary logistic regression in 4 designed models: Model. A: Not adjusted for variables; Model. B: Adjusted for age, sex, and BMI; Model. C: further adjustment for diabetes‐related variables (diabetes duration, HbA1C, and FBS); and Model. D was adjusted for model C variables as well as active smoking, alcohol consumption, hypertension status, TC, HLD, LDL, TG, eGFR, uric acid, albumin, creatinine and albuminuria. Statistical significance has been defined as a two‐sided alpha less than 0.05. SPSS version 26.0 has been employed for all statistical evaluations (SPSS).

## 3 RESULTS

### 3.1 Demographic and clinical characteristics of the cohort

Table.1 demonstrates the baseline characteristics of the study patients. The mean age of CAD and non‐CAD patients was similar between groups (54.8 and 54.7 years, respectively). Regarding the sex ratio, male patients made up 57.1% of the CAD group and 47.2% of the non‐CAD group (*p* = .12). The BMI (*p* = .01) and history of smoking (*p* = .04) were also greater in the CAD group, while the history of alcohol consumption did not show a contrast between the two groups. Regarding the clinical history, prevalent hypertension (*p* = .01) and the duration of diabetes (44.7 years vs. 42.8 years, *p* = .004) were grater among CAD patient. To compare the laboratory data, the significant characteristics were as follows: The levels of HDL and e‐GFR had a statistically lower mean in the CAD group, whereas the levels of HbA1C, TC, LDL, albuminuria and more importantly UCPCR all had a statistically higher mean in the CAD group (All with *p* < .05).

**TABLE 1:** Demographic, clinical and laboratory characteristic of studied type 1 diabetic patients categorized based on presence of CAD.

Variables	With CAD (*n* = 84)	Without CAD (*n* = 195)	*p*‐Value
Age, years	54.8 ± 5.7	54.7 ± 3.7	.811
Male, *n* (%)	48(57.1%)	92(47.2%)	.123
BMI, kg/m2	27.8 ± 4.3	25.9 ± 4.7	.012
Active smoking, *n* (%)	45(53.6%)	79(40.5%)	.043
Alcohol consumption, *n* (%)	13(15.5%)	26(13.3%)	.236
Hypertension, *n* (%)	46(54.8%)	70(35.9%)	.016
Diabetes duration, years	44.7 ± 4.0	42.8 ± 4.0	.004
HbA1C, %	8.6 ± 3.7	7.5 ± 4.2	.036
FBS, mg/dL	131.1 ± 5.8	130.1 ± 3.2	.066
TC, mmol/L	215.6 ± 8.5	200.1 ± 10.1	.007
HDL, mmol/L	45.4 ± 4.7	47.3 ± 6.7	.012
LDL, mmol/L	113.5 ± 5.1	111.1 ± 4.9	.001
TG, mmol/L	248.5 ± 12.9	247.1 ± 10	.367
Statin treatment, *n* (%)	72 (85.7%)	179 (91.8%)	.198
eGFR, mL/min/1.73 m^2^	87.4 ± 17.6	96.7 ± 19.7	.008
Uric acid, mg/dL	6 ± 2	5.5 ± 2.8	.123
Albumin, g/dL	3.8 ± 1.5	4.2 ± 1.9	.163
Creatinine, mg/dL	1.4 ± 1.1	1.3 ± 1.2	.569
Albuminuria, *n* (%)	25(29.8%)	37(18.9%)	.040
UCPCR, nmol/mmol	0.07 (0.05–0.08)	0.04 (0.03–0.05)	.005

Abbreviations: ALT, Alanine Transaminase; AST, Aspartate Transaminase; CAD, Coronary Artery Disease; eGFR, Estimated Glomerular Filtration Rate; FBS, Fasting Blood Glucose; HbA1C, Haemoglobin A1C; HDL, High‐Density Lipoprotein; LDL, Low‐Density Lipoprotein; TC, Total Cholesterol; TG, Triglycerides; UCPCR, Urinary C‐Peptide Creatinine Ratio.

For a more precise assessment of the patients, we decided to evaluate and compare them separately based on whether or not they had BMI exceeding 30. Accordingly, as shown in Table.2, certain characteristics were found to have significant differences between CAD and non‐CAD cases, when grouped based on being obese or not. In fact, similar to Table 1, variables including diabetes mellitus (years), TC, eGFR and UCPCR levels all showed statistically significant contrast between CAD and non‐CAD patients regardless of their BMI.

**TABLE 2:** Demographic, clinical and laboratory characteristics of studied type 1 diabetic patients based on presence of CAD and BMI.

Variables	Non‐obese (BMI < 30 kg/m^2^) (*n* = 106)			Obese (BMI ≥30 kg/m^2^) (*n* = 173)		
	With CAD (*n* = 37)	Without CAD (*n* = 69)	*p*‐Value	With CAD (*n* = 47)	Without CAD (*n* = 126)	*p*‐Value
Age, years	46.8 ± 6.1	44.9 ± 5	.188	43.2 ± 4.9	44.7 ± 2.7	.047
Male, *n* (%)	20 (54%)	34 (49.3%)	.656	28 (59.6%)	58 (46%)	.123
Active smoking, *n* (%)	16 (43.2%)	15 (21.7%)	.026	29 (61.7%)	64 (50.8%)	.263
Alcohol consumption, *n* (%)	5 (13.5%)	7 (10.1%)	.636	8 (17%)	19 (15%)	.796
Hypertension, *n* (%)	19(51.3%)	24(34.8%)	.112	27(57.4%)	46(36.5%)	.015
Diabetes duration, years	35.9 ± 4.8	32.4 ± 4.2	.002	34.1 ± 3.2	32.9 ± 3.5	.001
HbA1C, %	8.5 ± 4.5	7.2 ± 3.6	.126	8.7 ± 3.1	7.7 ± 4.5	.161
FBS, mg/dL	130.5 ± 6.4	129 ± 3.2	.166	131.5 ± 5.3	130.7 ± 3.1	.233
TC, mmol/L	216 ± 7.3	203.5 ± 9	.003	215.3 ± 9.5	198.3 ± 10.3	.001
HDL, mmol/L	46.2 ± 4	48.1 ± 3.7	.032	44.7 ± 5.1	46.9 ± 7.9	.126
LDL, mmol/L	112.4 ± 2.6	108.5 ± 3.5	.001	114.3 ± 6.3	112.6 ± 5.0	.196
TG, mmol/L	246.3 ± 11.4	244.6 ± 7.8	.496	250.3 ± 13.9	248.4 ± 10.9	.333
Statin treatment, *n* (%)	33(89.2%)	64(92.7%)	.441	39(82.9%)	115(91.3%)	.145
eGFR, mL/min/1.73 m^2^	86.3 ± 19.3	95.2 ± 18.6	.023	88.3 ± 16.4	97.6 ± 20.3	.025
Uric acid, mg/dL	6.2 ± 1.9	5.4 ± 2.1	.196	5.9 ± 2.1	5.5 ± 3.1	.410
Albumin, g/dL	3.7 ± 1.3	4.2 ± 1.5	.188	3.9 ± 1.6	4.2 ± 2.1	.452
Creatinine, mg/dL	1.4 ± 1.1	1.2 ± 1.1	.496	1.4 ± 1.2	1.4 ± 1.2	.563
Albuminuria, *n* (%)	12(32.4%)	17(24.6%)	.469	13(27.6%)	20(15.9%)	.814
UCPCR, nmol/mmol	0.06 (0.04–0.07)	0.04 (0.02–0.05)	.003	0.08 (0.05–0.10)	0.04 (0.03–0.05)	.006

Abbreviations: ALT, Alanine Transaminase; AST, Aspartate Transaminase; BMI, body mass index; CAD, Coronary Artery Disease; eGFR, Estimated Glomerular Filtration Rate; FBS, Fasting Blood Glucose; HbA1C, Haemoglobin A1C; HDL, High‐Density Lipoprotein; LDL, Low‐Density Lipoprotein; TC, Total Cholesterol; TG, Triglycerides; UCPCR, Urinary C‐Peptide Creatinine Ratio.

### 3.2 UCPCR as a predictor of CAD

Table.3 illustrates binary logistic regression on the possible risk factors of CAD among patients with T1DM. Model A calculates while not being adjusted for any of the variables, whereas Models B, C and D calculate the logistic regression while being adjusted for confounding variables. As it is exhibited in the Model D section, which exemplifies the most full‐scale assessment, among studied variables only some resulted in statistically significant outcomes including LDL, hypertension, HbA1C and UCPCR. Subsequently, the amount of odds ratio (OR) obtained for these variables could be interpreted as follows; in the case of LDL, it was found that individuals with elevated LDL levels were 2.9 times more likely to develop CAD (*p*‐value = .000); in the case of hypertension, it was discovered that patients who had a history of hypertension were 2.1 times more likely to have developed CAD (*p*‐value = .002); in the case of HbA1C, it was found that elevated levels of HbA1C could cause a 30% increase in the risk of CAD (*p*‐Value = .044); and finally in the case of UCPCR, it was indicated that increased levels of UCPCR could elevate the risk of CAD for 50% (*p*‐Value = .009).

**TABLE 3:** Binary logistic regression analysis for risk factors of CAD among patients with type 1 diabetes mellitus.

Variables	Model A		Model B		Model C		Model D	
	OR (CI 95%)	*p*‐Value	OR (CI 95%)	*p*‐Value	OR (CI 95%)	*p*‐Value	OR (CI 95%)	*p*‐Value
UCPCR	1.624 (1.224–2.156)	.038	1.497 (1.130–1.981)	.016	1.646 (1.2362–2.193)	.020	1.539 (1.155–2.053)	.009
Age	‐	‐	1.235 (0.877–1.741)	.225	1.170 (0.831–1.648)	.361	1.000 (0.706–1.421)	.992
Sex (male)	‐	‐	0.812 (0.569–1.158)	.356	0.763 (0.541–1.077)	.122	0.825 (0.587–1.160)	.274
BMI	‐	‐	1.545 (1.067–2.172)	.018	1.504 (1.069–2.117)	.011	1.354 (0.957–1.917)	.083
Diabetes duration	‐	‐	‐	‐	1.379 (0.893–1.914)	.546	1.237 (0.895–0.711)	.197
HbA1C	‐	‐	‐	‐	1.680 (1.188–2.375)	.003	1.307 (1.007–1.696)	.044
FBS	‐	‐	‐	‐	1.437 (1.011–2.044)	.043	1.372 (0.962–1.946)	.072
Active smoking	‐	‐	‐	‐	‐	‐	1.372 (0.967–1.946)	.093
Alcohol	‐	‐	‐	‐	‐	‐	1.103 (0.640–2.764)	.833
Hypertension	‐	‐	‐	‐	‐	‐	2.112 (1.298–3.435)	.002
LDL	‐	‐	‐	‐	‐	‐	2.859 (1.717–4.760)	.000
TG	‐	‐	‐	‐	‐	‐	1.475 (0.859–2.532)	.152
eGFR	‐	‐	‐	‐	‐	‐	1.775 (0.882–2.378)	.386
Uric acid	‐	‐	‐	‐	‐	‐	1.400 (0.916–1.843)	.125
Albumin	‐	‐	‐	‐	‐	‐	2.287 (0.999–3.020)	.125
Creatinine	‐	‐	‐	‐	‐	‐	1.985 (0.913–2.613)	.081
Albuminuria	‐	‐	‐	‐	‐	‐	1.410 (0.942–1.840)	.147

*Note*: Model. A: Not adjusted for variables. Model. B: Adjusted for age, sex and BMI variables. Model. C: Adjusted for age, sex, BMI and diabetes related variables (diabetes duration, HbA1C and FBS). Model. D: Adjusted for age, sex, BMI diabetes duration, HbA1C, FBS, active smoking, alcohol consumption, hypertension, TC, HLD, LDL, TG, eGFR, uric acid, albumin, creatinine and albuminuria.

Abbreviations: ALT, Alanine Transaminase; AST, Aspartate Transaminase; BMI, body mass index; CAD, Coronary Artery Disease; CI, confidence interval; eGFR, Estimated Glomerular Filtration Rate; FBS, Fasting Blood Glucose; HbA1C, Haemoglobin A1C; HDL, High‐Density Lipoprotein; LDL, Low‐Density Lipoprotein; OR, odds ratio; TC, Total Cholesterol; TG, Triglycerides; UCPCR, Urinary C‐Peptide Creatinine Ratio.

As the focal point of our study is to assess the effect of UCPCR, Table.4 demonstrates particularly the ORs of UCPCR for different logistic regression models and then compares them based on the BMI of patients. This analysis not only showed that elevated UCPCR values would significantly correlate with increased risk of CAD regardless of the age, sex, BMI and other well‐acknowledged CAD risk factors, but also it has shown that in each model, the OR for UCPCR is significantly rather greater for those with BMI ≥30 than it is for those with a BMI <30.

**TABLE 4:** Binary logistic regression analysis for association of UCPCR to CAD among patients with type 1 diabetes mellitus based on BMI.

Model	Non‐obese (BMI < 30 kg/m^2^)		Obese (BMI ≥30 kg/m^2^)	
	OR (CI 95%)	*p*‐Value	OR (CI 95%)	*p*‐Value
Model A	1.489 (1.223–2.329)	.035	1.576 (1.136–2.188)	.027
Model B	1.469 (1.076–2.062)	.017	1.471 (1.064–2.035)	.030
Model C	1.663 (1.279–2.430)	.038	1.705 (1.155–2.230)	.036
Model D	1.500 (1.091–2.062)	.022	1.526 (1.081–2.071)	.009

*Note*: Model. A: Not adjusted for variables. Model. B: Adjusted for age, sex and BMI variables. Model. C: Adjusted for age, sex, BMI and diabetes related variables (diabetes duration, HbA1C and FBS). Model. D: Adjusted for age, sex, BMI, diabetes duration, HbA1C, FBS, active smoking, alcohol consumption, hypertension, TC, HLD, LDL, TG, eGFR, uric acid, albumin, creatinine and albuminuria.

Abbreviations: BMI, body mass index; CI, confidence interval; OR, odds ratio.

## 4 DISCUSSION

In the present study, we have attempted to assess UCPCR levels in CAD patients and figure out whether CAD could be predicted in patients based on their UCPCR levels. Our analysis showed that UCPCR levels were significantly greater in CAD patients as a whole, and as sub‐grouped based on having a BMI ≥30. Also, further modellings conducted on the patients indicated that CAD cases, when adjusted for a variety of different variables, still had remarkably greater levels of UCPCR, regardless of their BMI.

Aggravation of systematic inflammation,^23^ oxidative stress condition,^24^ tissue hypoxia,^25^ microcirculatory damage^26^ and as a result, the formation of atherosclerotic plaques in T1DM may be the cause of the co‐occurrence of CAD. Nonetheless, in patients with T1DM, the involvement of various variables other than the mentioned well‐acknowledge risk factors for CAD has yet to be discovered. T1DM is recognized by autoimmune demolition of pancreatic beta cells. Recently, studies have shown that this destruction is not an ongoing process since some individuals with long‐standing T1DM still have functional residual pancreatic beta cells that produce C‐peptide as a co‐product of insulin biosynthesis.^27^, ^28^, ^29^ It has been long believing that C‐peptide lacks biological function, but recent studies have found that it has a variety of roles in various types of cells, despite the fact that its receptor is unknown. These activities include proliferation of smooth muscle cells, endothelial cells, mesangial cells and chondrocytes, chemotaxis of inflammatory cells involved in atherosclerotic plaque formation, accumulation in the sub‐endothelium and intima of vessel walls and pro‐inflammatory activities within mice's vascular cells.^30^ In lung capillary endothelial cells of mice, this is mediated via stimulation of the p38 protein kinase cascade, engagement of Na+/K + ‐ATPase in the renal tubule segment, and attachment to the G‐protein‐coupled receptors of Swiss 3 T3 fibroblasts. Also, studies showed that in hyperglycaemia, C‐peptide protects endothelium from apoptosis by inhibition of transglutaminase 2.^31^

Moreover, clinically, it was revealed that C‐peptide is related to a decrease in glomerular hyperfiltration and proteinuria, leading to increased risk of nephropathy. Also, low C‐peptide levels were correlated with greater glucose level variability, increased HbA1c, poor glycaemic control lower and as a result increased risk of retinopathy, neuropathy, foot ulcers and mortality.^32^, ^33^, ^34^, ^35^, ^36^, ^37^ On the contrary, higher levels of C‐peptide, as an indicator of insulin resistance, had been shown to relate to cardiovascular and all‐cause mortality in individuals without hyperglycaemia and with T1DM. Also, in a study by de Leon et al., they concluded that elevated C‐peptide levels predict coronary events earlier than impaired fasting glucose levels.^14^ These studies raise the conclusion that higher C‐peptide levels have been correlated with macrovascular complications and lower C‐peptide levels have been attributed to microvascular complications.^7^, ^38^, ^39^, ^40^, ^41^ Accordingly, a study by Wang et.al on 1299 Chinese population with T2DM, UCPCR levels was positively correlated with an increased risk of CAD (OR = 1.3; 95% CI = 1.0–1.6) and nephropathy‐related diabetes (OR = 1.1; 95% CI = 1.0–1.4).^42^

In this study, we compared CAD and non‐CAD patients based on the clinical and laboratorial characteristics and also categorized by BMI. We also considered well‐acknowledged risk factors of CAD including hypertension, dyslipidaemia, treatment with statins, mild to moderate reduced eGFR, diabetes‐related factors and sociodemographic properties. Similar to previous studies, those with CAD had significantly higher incidence of hypertension, albuminuria, TC, LDL, HbA1C, longer T1DM duration, as well as declined levels of eGFR and HDL. It is noteworthy that treatment with statins was generally high (>85% in both groups) in the participants of this study; thus, its effect on lowering the risk of coronary artery disease may have been diluted. This might explain why there is not a clear link between statins and coronary artery disease in this study.

A considerable number of diabetic individuals are obese with elevated BMI as the independent risk factor of CAD. Moreover, urinary C‐peptide, levels of C‐peptide and UCPCR could be influenced by weight. Therefore, following investigating the role of UCPCR on clinical CAD, we decided to analyse whether UCPCR have different effect in different BMI groups of T1DM patients. We obtained that among obese (BMI≥30) as well as the non‐obese (BMI < 30) group, UCPCR was related to CAD regardless of conventional CAD risk factors. These findings imply that UCPCR may be linked to or implicated in the production of atherosclerotic plaques within the coronary arteries of T1DM patients, both obese and non‐obese. Herein, UCPCR was suggested as a risk factor of CAD with the adjusted OR of more than 1, greater in non‐obese patients in comparison with obese patients. This may suggest the greater implication of UCPCR measurement in non‐obese patients.

One of the reasons for the importance of this study is that measuring UCPCR is simple, cost‐effective and practical in comparison with serum assays of C‐peptide and insulin.^43^ To the most precise of our knowledge, we have been the first to assess UCPCR as a risk factor of CAD in T1DM patients, classified by BMI. Nonetheless, there may be some limitations to the results of the present study. First, the present study has been a cross‐sectional, single‐centre one with limited study group size. Second, the diagnosis of CAD was mostly established by the patients' history and previous medical records. Third, patients had long‐lasting T1DM and were under treatment, even individuals with poor disease control. Forth, we were unable to investigate the chronological link between the UCPCR levels and the progression of coronary atherosclerosis due to the cross‐sectional nature of methodology; consequently, more longitudinal investigations are required. Fifth, due to lower power of analysis, we were not able to observe the impact of UCPCR based on more BMI categorization (i.e. underweight, normal‐weight and overweight BMIs). Sixth, other inflammatory factors known to be linked to cardiovascular risk (such as E‐selectin, C‐reactive protein, interleukin‐6 and intercellular adhesion molecule) have not been assessed, which may have resulted in a lack of explanatory power for C‐role peptides as the atypical inflammatory marker of CAD. Seventh, we excluded patients with eGFRs of less than 45 mL/min/1.73 m2; however, it is plausible this exclusion decreased the potential of our study to recognize a correlation between CAD, diabetic nephropathy and UCPCR. Last but not least, since the individuals in this study have been drawn from a single facility, there were inevitable biases in patient selection, acquired data and confounding factors.

In conclusion, this is a novel topic regarding the predictive role of UCPCR in patients with T1DM with lack of previous research studies. UCPCR is a practical and non‐invasive biomarker that has shown its effectiveness in differentiating T1DM from other DM groups, guiding the individual's treatment regarding poor‐ or well‐controlled DM, and as we showed, the subsequent CV risk prediction. We discovered that in T1DM patients, whether obese or not, UCPCR is associated with clinical CAD, independent of established CAD risk variables such as insulin resistance and glycaemic control. Meanwhile, further studies are required to evaluate the expansion of clinical implications and cost‐effectiveness of this marker in both fields of medical management and biomedicine.

## PATIENT CONSENT STATEMENT

Written informed consent has been acquired from patients to publish this study according to the journal's patient consent policy. Moreover, the authors all declare that patients' confidentiality has been respected. (Ethics code: IR.SBMU.MSP.REC.1400.083).

## AUTHOR CONTRIBUTIONS

**Farzad Esmaeili Tarki:** Data curation (equal); methodology (equal); project administration (equal); resources (equal). **Fatemehsadat Rahimi:** Data curation (equal); methodology (equal); writing – original draft (equal). **Nasser Malekpour Alamadari:** Conceptualization (equal); project administration (equal); supervision (equal).

## FUNDING INFORMATION

There is no funding to present study.

## CONFLICT OF INTEREST STATEMENT

The authors declare no conflict of interest.

## ETHICS APPROVAL STATEMENT

The authors all declare that this manuscript is not published elsewhere.

## Data Availability

The data supporting the findings of the study are available on request from the corresponding author.
